# Have the Olympic Games become more migratory? A comparative historical perspective

**DOI:** 10.1186/s40878-017-0054-2

**Published:** 2017-07-12

**Authors:** Joost Jansen, Godfried Engbersen

**Affiliations:** Department of Public Administration and Sociology, Erasmus University Rotterdam, PO Box 1738, 3000 DR Rotterdam, Burgemeester Oudlaan 50, 3062 PA Rotterdam, The Netherlands

**Keywords:** Olympic Games, Migration patterns, Athletic migration, Globalisation, Olympic citizenship

## Abstract

It is often believed that the Olympic Games have become more migratory. The number of Olympic athletes representing countries in which they weren’t born is thought to be on the rise. It should, however, be noted that migration in the context of sports is hardly a new phenomenon. In this paper we hypothesise that, as a reflection of global migration patterns and trends, the number of foreign-born Olympians hasn’t necessarily increased in all countries. Furthermore, it was expected that the direction of Olympic migration has changed and that foreign athletes increasingly come from a more diverse palette of countries. We conducted an analysis of approximately 40,000 participants from 11 countries who participated in the Summer Games between 1948 and 2012. The selected countries have different histories of migration and cover the distinction between ‘nations of immigrants’ (Australia, Canada, United States), ‘countries of immigration’ (France, Great Britain, Netherlands, Sweden), ‘latecomers to immigration’ (Italy, Spain) and, what we coin, ‘former countries of immigration’ (Argentina, Brazil). We conclude that the Olympic Games indeed have not become inherently more migratory. Rather, the direction of Olympic migration has changed and most teams have become more diverse. Olympic migration is thus primarily a reflection of global migration patterns instead of a discontinuity with the past.

## Introduction

In anticipation of the Rio 2016 Olympic Games, the International Organisation for Migration (IOM) published an online blog mentioning the fact that of the 558 athletes representing the United States, an “astonishing 44 foreign-born athletes will be donning the stars and stripes” (Ekin, 2016). These 44 athletes were born in 28 different countries, indicating the alleged super-diversity that marks our globalised era. Similarly, in 2012 the British tabloid newspaper Daily Mail reported that 61 ‘plastic Brits’ competed for Team Great Britain during that year’s London Olympics (Daily Mail 2012). The supposed increase in (the diversity of) immigrant Olympic athletes is often the subject of media controversies. Various stakeholders, especially international sports federations, call for measures to discourage nationality transfers and secure the nationalist character of the Olympic Games (Kostakopoulou & Schrauwen, 2014; Spiro, 2014). The very term ‘plastic Brits’ suggests that the ‘Britishness’ of these athletes is called into question. Are some British athletes more British than others? Sometimes, immigrant Olympians are even referred to as ‘Olympic mercenaries’: athletes willing to, without scruples, sell their talents to the highest bidding country (Kozlowska & Traywick, 2014). The examples raised in media discourses are often the same; be it a ‘Russian’ speed-track skater born in Korea or a ‘Qatari’ long distance runner from Kenya. Altogether, the common belief is that the Olympic Games have become increasingly migratory and diverse. It is perhaps not coincidental that Vertovec (2007) introduced his much-cited article on super-diversity by referring to the London bid to host the 2012 Olympics, which emphasised the similarities in terms of ‘multicultural diversity’ between the city itself and the Olympic Games, and of which Team Great Britain’s diversity might be the ultimate expression.

It should, however, be noted that migration in the context of sports is hardly a new phenomenon. Like many things in life it traces back to the ancient Greeks (Hardman & Iorwerth, 2012). During the Ancient Olympics mention was made of a talented Cretan long distance runner, named Sotades, who was bribed to become a citizen of and an athlete for Ephesus after first having competed and having won races for Crete. This evidently led to great Cretan discontent whereupon Sotades was banished from Crete (Kyle, 2015). Switching city-state allegiance to the highest bidding state was far from uncommon in those days. The question therefore is whether the assumption that Olympic teams increasingly consist of foreign-born athletes holds true. Hitherto such claims have not yet been subject to rigorous empirical testing. Migration within the context of sports is often merely a reflection of global and historical patterns of migration, rather than an isolated phenomenon. In the context of the migration of football players to the English Premier League, for instance, Taylor (2006, p. 7) concluded that “football migration is nothing new, but it has a long and complicated history; (...) it should not be isolated from general migratory trends and patterns.” Perhaps the same could be said about the number of foreigners representing Team USA or GB during recent editions of the Olympic Games. The purpose of this paper is to shed a comparative historical light on the “astonishing” number of foreign-born athletes who nowadays compete for other nations. To answer the question of whether the Olympic Games have become more migratory, we will analyse Olympic teams between 1948 and 2012. Through contrasting the results with broader migratory trends and patterns, we aim to place a common (mis)conception under scrutiny.

In the first part of this paper we discuss a conceptual framework based on research from both mainstream migration studies and the sociology of sport. This framework serves as a tool for comparatively and historically assessing how Olympic migration has evolved over time in terms of intensity, diversity, and direction. In the second part of this paper we discuss our methodological approach that follows from the theoretical framework. Lastly, we present the results of our analyses and elaborate on the implications and limitations of our study.

## “Has the world become more migratory?”

Whilst some academics state that we are now living in times of accelerating migration and super-diversity (cf. Castles, De Haas, & Miller, 2014; Vertovec, 2007), others contest this widespread belief. The idea that “the volume, diversity, geographical scope, and overall complexity of international migration have increased as part of globalisation processes remains largely untested” (Czaika & De Haas, 2014, p. 283). According to Czaika and De Haas this idea marks a Western bias or an Eurocentric worldview. Migration is not accelerating everywhere at the same pace (see also Flahaux & De Haas, 2016). Some traditional immigration countries (for instance Argentina and Brazil) are facing the opposite process: they have developed into countries of emigration. The authors conclude that under the unequal conditions of globalisation, migration has become increasingly non-European and less colonial (see also Penninx, 2016). Global migration has ‘skewed’ and ‘diversified’, but not necessarily increased everywhere. Throughout the twentieth century, the relative number of international migrants has remained quite stable, at about 3%. From a global perspective, the idea that we are now confronted with unprecedented migration seems to be flawed. It is therefore classified as one of seven common migration myths by De Haas (2005). However, it is true that international migration has become more visible. Recent imaginaries like the ‘migration crisis’ in Europe could explain why people tend to think of the world as becoming more migratory (De Haas, 2005; Goldin, Cameron, & Balarajan, 2012). This visibility-argument could also apply to the Olympic Games, as one of the greatest mediatised spectacles on the planet which is live broadcasted to over 200 countries in the world.

Over time countries have undergone different histories of migration (Castles et al., 2014). In this respect, Hollifield, Martin, and Orrenius (2014) differentiate between so-called ‘nations of immigrants’, ‘countries of immigration’, and ‘latecomers to immigration’. The first category applies to countries like the United States, Australia, and Canada. These are nations that have immigration as a part of their DNA, since they were established by immigrants. Countries like Great Britain, France, and the Netherlands belong to the second category. Although these countries have always been confronted with a vast influx of immigrants, they are hesitant in considering themselves countries of immigration. The third category applies to countries such as Italy and Spain. For a long time, they have accounted for a significant share of the world’s migration population. It was only during the last decades of the twentieth century that migratory movements to these countries began to increase. For the purpose of this paper, we propose to add a fourth category, which we call ‘former countries of immigration’ and applies to countries like Argentina and Brazil. They have evolved from countries of immigration into countries of emigration.

During the epoch of globalisation and super-diversity in which we are now thought to live immigration policies in immigration countries have become increasingly selective. Structural economic developments have changed the nature of labour markets, especially demanding more highly skilled workers (Castles et al., 2014; De Haas, Natter, & Vezzoli 2016; Hollifield et al., 2014; Penninx, 2016). The phenomenon of elite migration emerged, with countries competing for knowledge workers, sometimes even by offering them citizenship in exchange for their skills (De Haas et al., 2016; Shachar, 2006). This elite migration is not limited to regular highly skilled migration, i.e. the migration of lawyers, engineers, or academics. It has also expanded to the field of sports. In the hands of governments, migration is said to have become a tool with which countries try to enhance their global productivity, be it economic growth or the number of medals at the Olympics (Shachar, 2011).

Within the fields of sport sociology and history there’s an ongoing debate regarding whether or not athletic migration (as a form of elite migration) around the globe has intensified since the Second World War. Some authors (Bale & Maguire, 1994, p. 5) argue that, although nothing new “it appears, however, that the process is speeding up.” Most scholars sketch an increasing tendency of states taking an instrumental stance on the migration and naturalisation of talented athletes for state promotion purposes (cf. Poli, 2007; Maguire, 2011; Shachar, 2011). Against such notions, Taylor (2006) argues that the migration of athletes, *in casu* footballers, is not novel and can only be understood when related to general migratory trends and patterns. The movement of football players across the globe is merely a reflection and “adaptation of already existing patterns rather than any radical breach with the past” (Taylor, 2006, p. 30). Take for instance the post Second World War movements of footballers, such as Scottish players to the English leagues or the large influx of Argentinians and Yugoslavs since the 1970s. Similarly, after studying foreign footballers in the English football leagues, McGovern (2002, p. 23) concluded that notions of labour market globalisation are “fundamentally flawed, since they fail to account for the ways in which labour market behaviour is socially embedded.”

Taylor’s research focusses, like similar studies (Bale & Maguire, 1994), predominantly on movements of professional athletes across the world seeking employment elsewhere. The phenomenon that we address in this paper differs slightly from these movements in the sense that we focus on athletes who represent countries other than their own (rather than just ‘working’ in other countries). However, the main argument formulated in this paper is based on a combination of the above two elaborated arguments, taken from mainstream migration studies and studies on athletic migration. Firstly, that global migration has not intensified, but skewed and diversified and, secondly, that athletic migration is above all a reflection of global migration patterns. It is thus hypothesised that:

1. Migration within the context of the Olympic Games is above all an adaptation of already existing migration patterns and not so much a discontinuity with the past;
2. The number of foreign-born Olympians hasn’t necessarily increased in every participating country, but varies according to historical migration patterns. That means that, for instance, the number of foreign athletes in Italy and Spain is expected to grow over time, whilst the opposite applies to countries like Argentina and Brazil;
3. Foreign-born Olympic athletes increasingly come from a wide variety of countries of origin. The pool of foreign-born Olympians is thus expected to have become more diverse; and
4. The direction of the movement of Olympic athletes across borders has skewed. This implies that migration in the context of the Olympic Games has become increasingly non-European, less colonial, and more diverse.

## Methodology

To be able to map patterns of Olympic migration, one ideally needs detailed biographic data on every individual athlete of all participating countries. Subsequently, some measurement complexities arise. First of all, how to measure this specific type of migration? Many migration studies make use of foreign-born population data (see Castles et al., 2014; Dumont & Lemaître, 2005). But, as Horowitz and McDaniel (2015, p. 39) already noted, the validity of this country-of-birth metric is limited, since the “reason, timing and nature of an athlete’s move” remain obscure. We thus don’t know why and when an athlete migrated. An additional complexity results from the fact that IOC-regulations (as formulated in the Olympic Charter, chapter 5, rule 41) do not differentiate between various types of foreign athletes. Whereas in some countries foreign-born athletes are considered immigrants, in other countries they are seen as native athletes because they have acquired citizenship by descent (Brubaker, 1990; Dumont & Lemaître, 2005). The fact that the IOC until now does not openly register such detailed information (not even athletes’ birthplaces) thus impedes the central aim of this study. Additionally, national variations in citizenship legislation impose challenges to the aim of mapping historical patterns of Olympic migration.

The only feasible solution is, like Horowitz and McDaniel (2015), to use a foreign-born proxy and rely on secondary sources. Sports Reference LLC is the only known secondary source that provides information about the names and countries of birth of nearly all Olympic athletes since 1896. Unfortunately, also Sports Reference doesn’t provide complete data. For this study, it was therefore necessary to make a selection of a limited number of countries and editions (1948–2012). The eleven countries selected are (in alphabetical order) Argentina, Australia, Brazil, Canada, France, Great Britain, Italy, Netherlands, Spain, Sweden, and the United States. The motivation (both theoretic and pragmatic) for this selection is fourfold:

1. The selected countries have different histories of migration and thus together cover the distinction between ‘nations of immigrants’, ‘countries of immigration’, and ‘latecomers to immigration’ (Hollifield et al., 2014). For the purpose of this paper, we propose to add a fourth category, namely ‘former countries of immigration’ (Argentina and Brazil).
2. The selected countries employ different citizenship rules, based on either the principle of *jus soli* or *jus sanguinis* (or a hybrid form).
3. The selected countries participated in nearly all editions of the Summer Olympic Games after the Second World War, which allows us to map historical patterns.
4. Information on the birth countries of athletes from the selected countries is relatively complete compared to many other participating countries.

We constructed a dataset (see Jansen, 2017) by manually retrieving the names and countries of birth of all athletes in our selection from Sports Reference.^1^ The total dataset comprises over 40,000 participants. Some athletes were counted repeatedly as they participated at multiple editions, leaving us with approximately 29,000 unique athletes. In total, the country of birth is unknown for about 9% of the participants in the dataset, many of which are concentrated around the earlier editions (see Table 1, last column). We made some concessions regarding completeness (criterion 4), as, for instance, information on Argentinian and Brazilian athletes who participated in earlier editions is relatively incomplete. Nonetheless, we have chosen to select these countries based on our theoretical considerations (criterion 1).

**Table 1:** Share (%) foreign-born athletes by country by edition

	ARG	AUS	BRA	CAN	FRA	GBR	ITA	NLD	SPA	SWE	USA	TOT	UNK
1948	3.5%	2.7%	0.0%	5.5%	5.7%	10.2%	6.5%	10.7%	1.5%	1.7%	7.0%	6.2%	21.0%
1952	7.3%	1.2%	0.0%	4.7%	6.9%	4.3%	2.6%	14.4%	0.0%	1.5%	5.9%	4.8%	19.6%
1956	3.6%	6.5%	0.0%	4.3%	9.5%	5.3%	3.9%			4.5%	7.4%	6.0%	22.4%
1960	3.3%	11.6%	4.2%	18.8%	13.0%	8.3%	2.9%	8.2%	0.7%	4.5%	3.8%	6.9%	0.6%
1964	0.0%	7.9%	3.3%	13.9%	6.5%	5.9%	5.4%	7.2%	0.0%	1.1%	5.5%	5.8%	18.7%
1968	3.4%	6.3%	3.9%	23.7%	7.5%	8.4%	2.4%	6.5%	1.6%	5.0%	3.9%	6.6%	1.9%
1972	0.0%	5.4%	2.5%	20.7%	4.0%	6.0%	2.2%	7.6%	4.1%	4.6%	6.3%	6.3%	21.2%
1976	0.0%	5.0%	0.0%	19.5%	3.9%	9.5%	1.9%	4.6%	1.8%	5.2%	4.5%	7.1%	21.1%
1980	0.0%	5.8%	0.9%		6.6%	9.1%	1.3%	4.0%	2.6%	4.1%		4.3%	30.7%
1984		2.1%	0.0%	16.2%	4.6%	9.2%	2.6%	2.9%	0.6%	4.0%	6.1%	6.2%	13.0%
1988	0.8%	6.0%	0.0%	21.6%	6.0%	5.5%	2.4%	4.1%	3.9%	3.8%	4.4%	6.2%	12.8%
1992	1.2%	7.9%	1.1%	19.3%	8.8%	7.3%	3.0%	6.5%	3.3%	4.8%	5.3%	6.6%	7.8%
1996	0.6%	10.1%	1.8%	15.8%	8.6%	5.7%	2.9%	6.7%	5.9%	3.4%	6.3%	6.7%	3.8%
2000	2.1%	13.9%	1.0%	17.0%	8.0%	4.8%	8.0%	8.2%	5.3%	2.7%	5.3%	8.0%	1.5%
2004	1.3%	10.2%	2.1%	12.5%	11.4%	5.3%	10.7%	12.4%	5.7%	7.0%	5.4%	7.9%	0.0%
2008	2.3%	9.7%	1.1%	13.9%	7.1%	8.2%	7.2%	11.0%	8.5%	5.7%	6.1%	7.7%	0.0%
2012	2.9%	11.1%	2.0%	12.8%	9.3%	13.0%	8.5%	11.0%	10.1%	3.0%	8.1%	9.2%	0.0%

Source: Authors’ calculations

In terms of analysis, the historical patterns of Olympic migration are compared with global migration patterns. In their paper Czaika and De Haas (2014) test the common belief that the volume, diversity and scope of migration have globalised by conceptualising globalisation using several indicators, of which *intensity*, *diversity*, and *direction* are pivotal. Intensity is measured as the relative share of migrants as a percentage of a given population. They define diversity as the extent to which immigrants come from a variety of countries of origin. Diversity (*D*) is calculated by using the Herfindahl-index, a measure derived from economics (used to calculate market concentration). Other sociologists (cf. Putnam, 2007) have also adopted this measure to calculate ethnic diversity. For each country, it is used to calculate the sum of squares of the proportion of each immigrant population (*IM*_*i*_) as a share of the total immigrant population (*M*). An outcome close to 1 indicates relatively high diversity, whereas an outcome close to 0 indicates homogeneity.

$$ D=1-{\sum}_{i=1}{\left(\frac{IM_i}{M}\right)}^2 $$

Regarding direction, Czaika and De Haas (2014, p. 315) refer to the changing direction of contemporary global migration flows: “Migrants from an increasingly diverse array of non-European-origin countries have been concentrating in a shrinking pool of prime destination countries.” In this paper, we compare Olympic migration and global migration patterns on these three indicators in order to determine whether the Olympic Games have become more migratory.

## Intensity

In previous decades, the relative share of migrants as a share of the total world population has remained relatively stable, at about 3%. In line with earlier remarks about this figure, not all countries have faced the exact same patterns of immigration since the Second World War. Three major transitions can be discerned (Czaika & De Haas, 2014). First of all, South American countries like Argentina and Brazil were among the top immigration destinations before and during the 1960’s, but gradually migration to these countries has reduced. Contrarily, some European countries are facing the opposite process. During the second half of the twentieth century, migration patterns reversed as countries like Italy and Spain changed from emigration to immigration countries. European migrants now account for a relatively small share of the global migrant population. Other European countries (think of France, Great Britain, and the Netherlands) have entered the immigration phase somewhat earlier, partly due to their colonial histories and partly due to the recruitment of guest workers. Thirdly, traditional ‘nations of immigrants’ like the United States and Australia have always attracted many immigrants, just as they do nowadays.

Applying these findings to Olympic migration, it was expected (in line with the second hypothesis) that the number of foreign-born athletes wouldn’t necessarily have increased in all participating countries. Table 1 shows that this indeed seems to be the case for the 11 countries selected in this study. The overall share of foreign-born athletes has only slightly increased over the past 60 years and fluctuates between roughly 4 and 9%. Regarding this fluctuating increase we need to make two important remarks. First, we need to take into account that there is considerable uncertainty regarding the birth countries of about 20% of the athletes participating in earlier editions. If we were to extrapolate, for instance, the total share of 6.1% foreign-born athletes in 1948, we would estimate it at roughly 8%. But, to avoid errors (mistakenly counting native athletes as foreign-born), we have chosen to employ a conservative approach and solely base our analyses on the information that is certain (Horowitz & McDaniel, 2015; Oettl & Agrawal, 2008). Our second remark concerns the overall proportion of foreign-born athletes, which turns out to be higher than 3% (the proportion of migrants as a share of the total world population). Given our selection of high-profile migration countries, this deviation seems logical and maybe even somewhat small. In 2014 for instance, the percentage of foreign-born persons in OECD countries was 13% (OECD, 2016). At the Olympic Games of 2012, the total share of foreign-born athletes was about 9%. Based on research on global migration trends and patterns we a priori expected their share to be higher than 3%.

As hypothesised, during earlier editions of the Olympic Games the three ‘nations of immigrants’ (Australia, Canada, and the United States) have always been represented by a significant number of foreign-born athletes. At the Mexico 1968 Summer Olympics, Team Canada (139 athletes) was represented by no less than 33 foreigners (nearly 24%), accounting for the highest share of foreign-born athletes of all observed countries since 1948. Gradually, their numbers declined. In 2012, Canada was represented by 35 foreign-born athletes (nearly 13%). Although not as high, the same applies to Australia. At the 1960 games held in Rome, 22 of 189 athletes weren’t born in Australia. In 2012, this was the case for 45 athletes out of a total of 404. In both editions, the relative share of foreign-born athletes was about 11%. When Sydney hosted the Summer Olympics in 2000, 86 Australian athletes (14%) were born abroad. This is the highest absolute number of foreign-born Olympians in our dataset. The United States, often referred to as one of the countries that nowadays sees no shame in capitalising athletes from abroad (Shachar, 2011), was represented by 22 foreign-born athletes in 1956 (about 7.5%). In 2012, their numbers were 43, which prima facie seems to be a lot. Their relative share however just exceeds 8% of the whole team.

Argentina and Brazil are well-known for once welcoming large numbers of immigrants. However, over the past decades this influx stagnated. This pattern then should be reflected in the number of foreign-born athletes representing these two countries. Although there is much more uncertainty regarding the birth countries of some of their athletes (especially during earlier editions), it seems that the expected pattern arises. In 1960, four out of 72 athletes competing for Brazil were born abroad. We counted zero foreign athletes competing for Brazil in the editions before 1960. This might be partly explained by the high percentages of athletes whose countries of birth are unknown (varying from 50 to 60%). In 2012, the share of foreign-born athletes declined to just 2% (5/248). Similarly, in 1952 about 7% of the Argentinian athletes were born abroad. In 1948 and 1956, the share of foreign athletes was somewhat lower at 3.5 and 3.6% respectively. However, uncertainty regarding the birthplaces of Argentinian athletes participating in those editions was also significantly higher (resp. 49 and 54%, compared to 39% in 1952). In London, the share of foreign-born Argentinian athletes was only 2% (with zero uncertainty).

As for the typical countries of immigration (France, Great Britain, Netherlands, and Sweden) we expected that, due to their histories of migration, they have always been represented by lots of foreign athletes, especially those with colonial backgrounds. Their numbers are likely to have increased since the 1990s when the European Union (EU) became a prime migration destination. Yet again, the expected pattern emerges. When Great Britain hosted the 1948 Summer Olympics, nearly 10% of their athletes (40) were foreign-born. A figure that places the 66 foreigners (12.5%) in the London 2012 Olympics in a different light. The share of foreign-born British athletes seems to concord with general immigration trends in the UK. The gradual increase in foreign athletes between 2004 and 2012 appears to have taken place slightly faster than, yet consistent with, a gradually increasing stock of foreign-born people in the UK (OECD, 2016). The consistency between OECD stock-data and our data also applies to France and the Netherlands. Although the stocks of foreign-born athletes have fluctuated from edition to edition (especially in France), we note an upward trend line from the 1980s onwards. Sweden proves to be a somewhat different case compared to the other countries in our dataset, for the relative share of foreign-born athletes has often varied and remained quite low compared to national immigration rates. Lastly, as latecomers to immigration, Italy and Spain have witnessed an overall increase of athletes born abroad, which is also consistent with general immigration trends. In 2012, their teams were composed of 8.5 and 10%, respectively, foreign-born athletes.

## Diversity

Having demonstrated that the Summer Olympics have not become inherently more migratory, we come to the third hypothesis: the pool of foreign-born athletes is becoming increasingly diverse. To verify this, we calculated the diversity among foreign athletes using the Herfindahl-index. We have chosen to compare 1960 and 2012 because information on both editions is near complete and it allows us to contrast the outcomes with global immigration diversity (cf. Czaika & De Haas, 2014). An outcome close to 1 indicates high diversity, whereas an outcome close to 0 indicates concentration.

Table 2 shows that foreign-born Olympic athletes increasingly come from a wide range of different sending countries. The outcomes indicate that Olympic migration is diversifying. Again, this seems to be a reflection of global migration patterns, rather than an isolated phenomenon. On a global scale, migration has also diversified, as immigrants increasingly originate from a wide array of sending countries all over the world.^2^ In earlier editions, there was less diversity among the origin of foreign-born athletes, as many of them had a European or colonial background (e.g. fencers from Hungary after the Hungarian revolution in 1956, British hockey players from India). Nowadays, foreign-born athletes come from all parts of the world. Caribbean migrants for instance now account for a substantial share of the global athletic migration (e.g. Jamaican runners competing for Canada).

**Table 2:** Diversity (D) among foreign-born athletes

	1960	2012
Argentina	0.444	0.625
Australia	0.860	0.935
Brazil	0.667	0.560
Canada	0.898	0.924
France	0.730	0.911
Great Britain	0.898	0.949
Italy	0.688	0.906
Netherlands	0.494	0.892
Spain (In 1960, only one athlete competing for Spain was born abroad, hence the outcome of zero diversity.)	0.000	0.923
Sweden	0.667	0.750
United States	0.860	0.962
All 11 countries	0.953	0.972

Source: Authors’ calculations

On a country level, it turns out that not all countries have become equally diverse, let alone at the same pace. If at all, it seems that South American countries are diversifying at a slower pace, a finding that is consistent with global migration statistics (Czaika & De Haas, 2014). In comparison to the other participating countries, foreign-born athletes competing for Great Britain, France, Canada and the United States have always come from a variety of countries. Other countries, like the Netherlands, were less diverse during earlier editions of the Olympic Games. In the case of the Netherlands, a relatively high influx of foreign-born athletes mainly stems from colonial linkages. Many foreign-born Dutch athletes that participated in the editions of 1948 and 1952 were born in Indonesia and had (by analysing the look of their names) Dutch roots. The Olympic teams of Great Britain and France, having had more colonies than the other countries in our selection, also consisted of a more diverse palette of foreigners. In 1948 and 1952, many French athletes born abroad came from Morocco and Algeria.

Our data indicate that colonial linkages aren’t as important as they used to be in explaining migration in the Olympic context, a shift that corresponds with global migration patterns. Czaika and De Haas (2014, p. 315) note that: “(…) migration from many developing and former colonies tended to be concentrated on the former colonisers (e.g., from the Maghreb countries to France; or from Guyana to Britain) because of economic, social, cultural, and linguistic ties. These ties may have eroded over time, possibly coinciding with a diversification of migration.” Similarly, Penninx (2016) argues that European immigration in the 1960s was to a significant extent determined by colonial ties, whereas nowadays the immigration in Europe is highly diversified. As we will demonstrate in the next section, Olympic migrants too increasingly come from different sending regions.

## Direction

In the remainder of this article, we will highlight two epochs of migration that occurred over the course of Olympic history (since the Second World War) through a country-of-origin perspective. These epochs are meant to illustrate the fourth hypothesis underpinning this article, namely that the direction of the movement of Olympic athletes across borders has skewed in the previous decades. During the first period after the Second World War, global migration patterns were predominantly European and to a large extent determined by colonial linkages. Nowadays, because of its skewed directional nature, migratory movements tend to be less European and more diverse in their offspring.

**Fig. 1:**
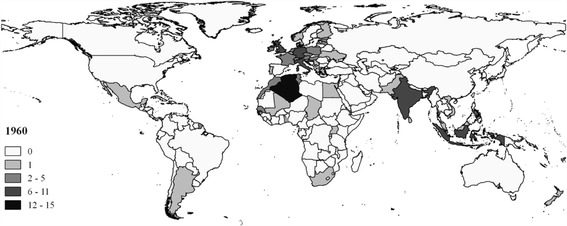
Absolute no. of foreign athletes by country of birth (1960). Source: Authors’ calculations and presentation

Table 3, which shows the cross-continental movements of foreign-born Olympic athletes in 1960, forms a perfect illustration of the first epoch after the Second World War. During the Rome 1960 Olympic Games, about 55% of the Olympians born abroad originated from Europe. Of all foreign athletes competing for both North American countries (Canada or the United States) and South American countries (Argentina and Brazil), more than 80% was born in Europe. Germany, Great Britain, Italy, and Croatia were among the top sending countries in terms of the absolute number of foreign-born athletes. Asia and Africa together accounted for about 38% of the share of foreign Olympians, which can be explained by the importance of colonial linkages at the time (with Algeria, Morocco, India, and Indonesia as important countries-of-origin). These two findings are perfectly illustrated in Fig. 1 (darkness indicates relatively high outflow), showing the predominantly European and colonial character of Olympic migration during the first epoch after the Second World War.

**Table 3:** Cross-continental movements of foreign-born Olympic athletes

1960	Destination continent				
Origin continent	Europe	North America	Oceania	South America	Total
Africa	37.0%	0.0%	0.0%	0.0%	21.1%
Asia	19.2%	7.4%	27.3%	0.0%	17.2%
Europe	35.6%	85.2%	72.7%	83.3%	54.7%
North America	8.2%	3.7%	0.0%	0.0%	5.5%
Oceania	0.0%	3.7%	0.0%	0.0%	0.8%
South America	0.0%	0.0%	0.0%	16.7%	0.8%

Source: Authors’ calculations

The London 2012 Olympic Games are noted for their multicultural character and hence form a perfect illustration of the second epoch in the modern history of Olympic migration: the epoch of diversity. In accordance with global migration patterns, we hypothesised that foreign-born Olympic athletes increasingly tend to come from non-European and non-colonial countries. Overall, Table 4 indeed shows the relative decline of foreign-born Olympians with a European background, resulting in a more equal distribution of foreign-born athletes over origin continents. Whereas in 1960 over 80% of the foreign athletes representing a North or South American country were born on the European continent, in 2012 their share has significantly decreased. Together, these figures indicate how the direction of cross-continental movements has changed over the decades.

**Table 4:** Cross-continental movements of foreign-born Olympic athletes

2012	Destination continent				
Origin continent	Europe	North America	Oceania	South America	Total
Africa	26.0%	10.7%	26.7%	0.0%	21.5%
Asia	8.7%	16.0%	26.7%	22.2%	13.6%
Europe	35.8%	38.7%	35.6%	44.4%	36.8%
North America	20.8%	26.7%	8.9%	33.3%	20.9%
Oceania	2.3%	2.7%	2.2%	0.0%	2.3%
South America	6.4%	5.3%	0.0%	0.0%	5.0%

Source: Authors’ calculations

**Fig. 2:**
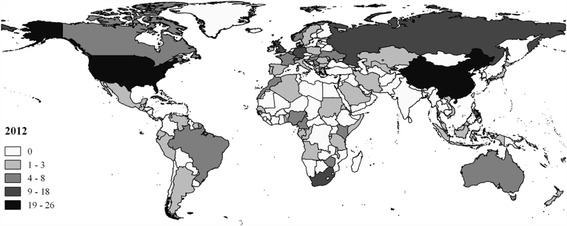
Absolute no. of foreign athletes by country of birth (2012). Source: Authors’ calculations and presentation

In total, the share of athletes from North America, Oceania and South America has grown quite significantly. New countries of emigration have emerged, like Cuba, Jamaica, China and Brazil. Between 2004 and 2012, 20 athletes born in Cuba represented either Canada, Great Britain, Spain or the United States. In that same period, there were 33 Chinese athletes competing for another country (almost all of them played badminton or table tennis). On a global scale, China also happens to be the country with most emigrants to OECD countries (OECD, 2016). Again, this illustrates the central argument of this paper: Olympic migration is above all a reflection of global migration patterns, and therefore our data seem to concord well with OECD data on international migration. The diversified and skewed directional nature of current migration flows is visualised in Fig. 2. Foreign athlete now come from a variety of countries all over the world. In comparison with Fig. 1, one can clearly see that, relative to other countries, fewer foreign athletes were born in (former) colonies.^3^

It must also be added that Olympic migrants often come from developed countries such as Germany, China, the United States and Great Britain. Although these countries are also among the top 15 countries of origin to new OECD countries (OECD, 2016), we might be dealing with a context-specific pattern here. Many athletes born in these countries face high competition in their home countries to be selected to participate at the Olympic Games. Hence, they might seek refuge elsewhere to chase their Olympic dreams. Because of the non-biographic nature of our data (no information on why and when an athlete has migrated) it is hard to verify such hypotheses.

## Conclusion and discussion

In contrast to what many people tend to believe, we have argued that the Olympic Games have not become “astonishingly” more migratory. We must be hesitant to conceive of our times as radically different from the past. Migration in the context of the Olympics is above all a reflection of global migration patterns. Our results indicate that in the history of the Olympic Games, the selected countries have always been represented by sizeable amounts of foreign-born athletes. Olympic migration during earlier editions can to a great extent be characterised as European and colonial. Nowadays, in the epoch of diversity, foreign-born athletes come from all corners of the world. Overall, the intensity, diversity, and direction of Olympic migration correspond with OECD statistics on global migration flows.

That is not to say that all countries are confronted with the same processes. It is important to note that countries have different histories of migration. Therefore, we need to distinguish between ‘nations of immigrants’, ‘countries of immigration’, ‘latecomers to immigration’ and, what we have coined, ‘former countries of immigration’ (Hollifield et al., 2014; Czaika & De Haas, 2014). Countries belonging to the first category (Australia, Canada, and the United States) have always been represented by many foreign-born athletes, especially those from Europe. However, the diversity among foreign-born athletes has grown significantly over the past editions of the Summer Olympics. The same can be said for countries belonging to the second category (France, Great Britain, and the Netherlands). In the period following World War II, these countries were often represented by athletes with a colonial background. Team Great Britain was composed of substantial numbers of athletes born in India, France was represented by many Moroccan and Algerian athletes, and the Netherlands had many athletes born in Indonesia among their ranks. Nowadays, like in Australia, Canada, and the United States, foreign-born athletes come from a wide array of sending countries. The third category applies to countries such as Italy and Spain. They have only recently entered a phase of immigration, a trend that is also reflected in Olympic context. Lastly, Czaika and De Haas (2014) have shown us that, as former immigration countries, Argentina and Brazil have developed into countries of emigration. Over the course of Olympic history, we have also observed a relative decline of foreign-born athletes representing these countries.

Two major points of debate arise from the findings that we have presented in our paper. First, our results suggest that, rather than a dramatic overall increase in foreign-born immigrants, it is the public perception on immigration that has changed over the past decades. It may very well be that Olympic migration has become more visible as a result of increased mediatisation and is therefore conceived of as more prevalent (cf. Czaika & De Haas, 2014). Another possible explanation for a change of public perception lies within the fact that although the number of foreign-born athletes (or immigrants in general) has not dramatically increased in all countries, second or third generation immigrants are sometimes considered to be immigrants too. In addition to the number of foreign-born athletes, a substantial share of the ‘native’ Olympic athletes in our database might have a migration background. We would argue that taking these ‘immigrants’ into account does not make a significant difference in terms of diversity or direction. However, it could lead one to perceive the Olympics as more migratory, especially in a context where nationalist backlashes have contributed to the reconstruction of immigrant-native boundaries along ethnic lines, causing second generation immigrants to (still) be perceived as immigrants (Alba, 2005; Goldin et al., 2012). When, for instance, looking at second or even third generation Moroccan footballers representing the Netherlands, Van Sterkenburg (2013) found that they, as a result of ‘conditional belonging’, are considered either Dutch or Moroccan depending on their sport performances. Given the limitations of our data it is hard to challenge such discourses.

A second point of debate concerns the complex issue of Olympic citizenship. Given the fact that IOC-regulations base Olympic nationality on an athlete’s citizenship status(es) and countries employ different citizenship laws, there are always situations in which athletes are entitled to represent different countries, because they have multiple citizenship. Moreover, because of these variations, a foreign-born athlete is not necessarily considered foreigner in every country. To overcome such complex citizenship issues that form an impediment to measuring and mapping international migration, the OECD (cf. Dumont & Lemaître, 2005; OECD, 2016) has started developing new databases that include information on both the birthplaces and nationalities of migrants. Dumont and Lemaître (2005) found that using foreign-born data generally leads to an overestimation of foreigners. In the Olympic context, this would imply that the share of foreign athletes in, for example, 2012 would be lower than 9.2%, because it is likely that many foreign-born athletes in our dataset are actually considered natives in the countries they represent (via the principle of *jus sanguinis*). With Horowitz and McDaniel (2015) we call upon the International Olympic Committee and other stakeholders to start registering more precise information about the nationalities of athletes. This will help future studies to map historical patterns of Olympic migration and citizenship changes in more detail. Moreover, such information would allow future research to verify the belief that, increasingly, countries are utilising migration as a means to enhance their Olympic performance and that athletes are easily swapping allegiance to the highest bidding country. Because of the nature of our data, it is difficult to verify such claims, as more detailed information (e.g. about the nationalities of athletes) is lacking. Yet, despite the limitations of our study in terms of data completeness and selectiveness, we have been able to generate new insights on global migration patterns in the context of the Olympic Games. By adopting a comparative historical perspective, we have thus tried to dispel the commonly accepted myth that the Olympic Games have become more migratory.

## Footnotes

1 As of mid-2017, the information provided by Sports Reference LLC (at www.sports-reference.com/olympics) will not be available any longer due to changing data licensing agreements between Sports Reference and their data providers. To ensure the sustainable archiving and public accessibility of the data on which we have based this paper, we submitted the dataset to the online data repository DANS EASY (an institute of the Royal Netherlands Academy of Arts and Sciences and the Netherlands Organisation for Scientific Research). The data can be accessed via: https://doi.org/10.17026/dans-2xf-pyqp.

2 For the years 1960 and 2000, Czaika and De Haas (2014) calculated a global immigration diversity of 0.980 and 0.993 respectively.

3 As a result of increased diversity, the distribution of foreign-born athletes over countries of birth differs notably between 1960 and 2012. Therefore, it was not possible to use the same legend for both figures without the loss of valuable information. Consequently, we have chosen to let QGIS set natural breaks for both figures. As a result, a comparison between Figs. 1 and 2 can only be made to illustrate the increased diversity and changed direction of Olympic migration.
